# Supplementary material for: Mechanically reconfigurable multi-functional meta-optics studied at microwave frequencies
Source: Sci Rep. 2021 May 27;11:11145. doi: 10.1038/s41598-021-88785-5 (PMC8160010; doi:10.1038/s41598-021-88785-5)
Supplement: Supplementary file 1 — Supplementary Information. [file 41598_2021_88785_MOESM1_ESM.docx]

**Supplementary Information**

**Mechanically reconfigurable multi-functional meta-optics studied at microwave frequencies**

Conner Ballew, Gregory Roberts, Sarah Camayd-Muñoz, Maximilien F. Debbas, and Andrei Faraon^*^

Kavli Nanoscience Institute and Thomas J. Watson Sr. Laboratory of Applied Physics, California Institute of Technology, Pasadena, California 91125, USA

*faraon@caltech.edu

# 1) Effective index of PLA slab in microwave cavity

For simplicity of measurement, the 3D-printed polylactide acid (PLA) devices have a thickness smaller than the height of the microwave cavity chamber. The microwave chamber height is approximately 8 mm while the devices are printed with a height of 6.4 mm, or 80% of the waveguide height. The effective index can be computed with eigenmode solvers. Here we use a mode source in Lumerical FDTD to compute the frequency-dependent effective index of the fundamental mode of the cavity. Note that the height of the cavity is chosen such that only this fundamental mode propagates, even when the cavity is fully loaded with PLA. The effective index as a function of the percentage of the cavity filled with PLA is plotted in Fig. S1. Individual lines are plotted for frequencies ranging from 7.6 GHz to 11.6 GHz, which shows some dispersion. This dispersion was not considered when optimizing the devices.


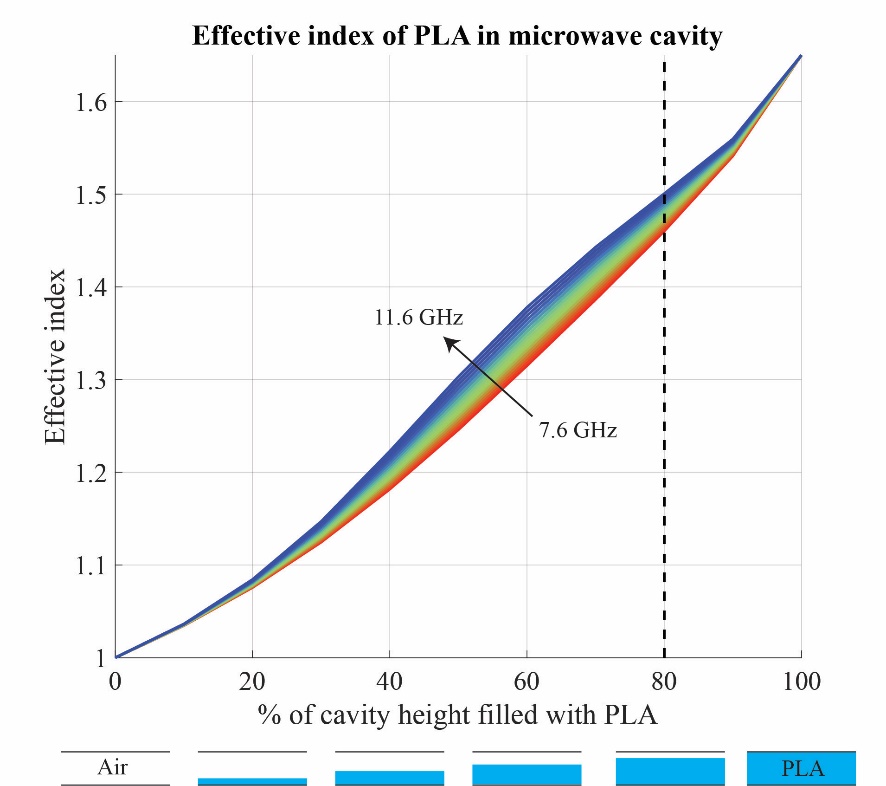


Figure S1: **Effective index of a PLA slab in the microwave cavity.** The colored lines represent the frequency, with red at 7.6 GHz and blue at 11.6 GHz. The approximate height of the designed PLA devices is 80% the cavity height, which is shown with the dashed black line.

# 2) Convergence plots

The design techniques used here are based on gradient descent, which incrementally steps the permittivity of the device in a way that is informed by the sensitivity of the various figures of merit with respect to device permittivity. Gradient descent techniques converge to a locally optimal point. In this density-based optimization, where the permittivity of the devices is modelled as a greyscale value between the permittivity of air and PLA, the optimization is not guaranteed to converge to a solution in which all voxels of the device are binary (either air or PLA).

To fix this, at each step of the optimization problem we solve a sub-optimization problem that seeks to maximize the change in performance of the device while constraining the permittivity step such that the device will become more binary. The effect of this is seen in the atypical convergence plot of the shearing device, which reaches peak performance when the binarization of the device is less than 100%. The remaining iterations push the overall binarization of the device towards 100% while seeking to maximally increase (or in this case minimally decrease) the performance.

To ensure the device is fully binary, the device undergoes a thresholding operation in which all voxels are rounded to the nearest permittivity boundary. Since the devices are nearly binary prior to this operation, this thresholding has a negligible effect on device performance.

*NOTE: During the optimization the figure-of-merit was tracked and stored using transmission through a smaller window (3 cm) than is presented in the main manuscript (6 cm). Thus, the reported average figure of merit value here is lower than the ones presented in the main manuscript.*


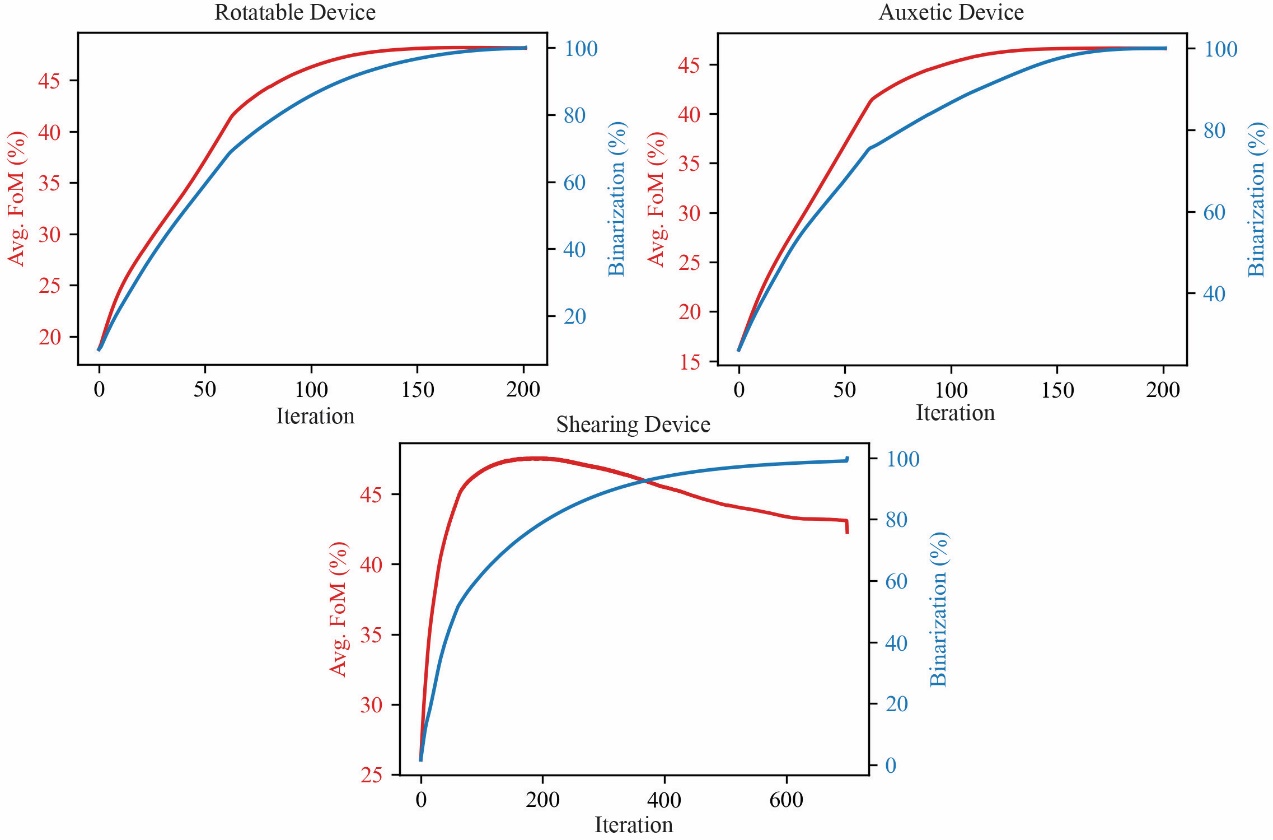


Figure S2: **Convergence plots of the different devices.** The red lines indicate the average figure of merit, and the blue lines represent the overall binarization of the device defined as $mean\left( |\frac{\boldsymbol{\epsilon}-\epsilon_{mid}}{\epsilon_{high}-\epsilon_{mid}}| \right)$, where $\boldsymbol{\epsilon}$ is a vector representing the permittivity of the device at each point, $\epsilon_{high}$ is the upper bound of permittivity, and $\epsilon_{mid}$ is the midpoint between the upper and lower permittivity bounds. On the final iteration, each device undergoes a thresholding operation that forces each voxel to its nearest material boundary. The performance is not substantially affected since the devices are nearly binary prior to the operation.

Supplementary Visualization I: **Demonstration of the transformation of 3D-printed squares.** The squares are 3D-printed simultaneously. Midway through the print, a Nylon mesh is placed on the device and the 3D print is resumed. The Nylon mesh is cut, leaving only the appropriate hinges.
